# Paternal intelligence affects school grades in children with and without ADHD - a register-based study

**DOI:** 10.1007/s00787-024-02510-x

**Published:** 2024-06-27

**Authors:** Andrea Markkula, Kajsa Igelström, He Zhang, Andrea Johansson Capusan

**Affiliations:** 1 Division of Psychiatrics & Rehabilitation & Diagnostics, Department of Child and Adolescent Psychiatry, Ryhov County Hospital, Jönköping, Sweden; 2 Department of Child and Adolescent Psychiatry in Linköping, Department of Biomedical and Clinical Sciences, Linköping University, Linköping, Sweden; 3 Division of Neurobiology, Department of Biomedical and Clinical Sciences, University Hospital Campus, Linköping University, Linköping, Sweden; 4 Faculty of Medicine and Health Sciences, Forum Östergötland, Linköping University, Linköping, Sweden; 5 Center for Social and Affective Neuroscience, Department of Biomedical and Clinical Sciences, Linköping University, Linköping, Sweden; 6 Department of Psychiatry in Linköping, Department of Biomedical and Clinical Sciences, Linköping University, Linköping, Sweden

**Keywords:** ADHD, School grades, Paternal IQ, School performance

## Abstract

ADHD profoundly impacts educational attainment, quality of life, and health in young adults. However, certain subgroups of ADHD patients seem to do quite well, potentially due to differences in intelligence and socioeconomic status. Here we used paternal intelligence from the Swedish Defence Conscription and Assessment register, to investigate the role of genetic propensity for intelligence, on school performance in a large cohort of ADHD patients and matched controls. Patients treated for ADHD in Linköping, Sweden between 1995 and 2020 (*n* = 3262), sex- and age-matched controls (*n* = 9591) as well as their parents and siblings were identified using regional and national registers. Socioeconomic and demographic data, ADHD diagnosis and treatment and school grades at age 16 for the study population were extracted from Swedish National registers. We explored the associations between paternal intelligence and child school performance using linear mixed models and mediation analyses, taking a wide range of potential covariates into account. Results indicate that paternal intelligence was positively associated with standardized school grades in their offspring (Z_adjusted_=0.09, 95%CI 0.07, 0.10). This effect was present in both ADHD patients and controls, but ADHD patients had significantly lower standardized grades (Z_adjusted_=-1.03, 95%CI -1.08, -0.98). Child ADHD did not serve as a mediator for how paternal intelligence affected school grades. Our findings indicate that ADHD prevents children from reaching their academic potential at all levels of paternal intelligence. Increased understanding of the contributions of ADHD, intelligence, and SES to functional outcomes can help clinicians to better personalize interventions to the unique preconditions in each patient.

## Introduction

Attention deficit hyperactivity disorder (ADHD) as well as ADHD symptoms in childhood and adolescence impact school grades, education, quality of life, and health in young adults [1–4]. Academic problems are common in ADHD, start early in life and are often the reason for children to come to clinical attention [1]. ADHD medication seems to be associated with improved school grades among ADHD patients in some [4] but not in all studies [5, 6]. Interestingly, among both medicated and unmedicated ADHD patients, some subgroups seem to do quite well academically [6, 7]. In a previous study, 20% of adolescent ADHD patients were considered well-functioning in several domains such as school and social function [8]. There are indications that a high IQ, or general intelligence, may compensate for ADHD related problems [9].

Emerging evidence suggests that intelligence may serve as the most comprehensive predictor of life trajectories as measured in educational and occupational performance, socio-economic status (SES), health, and longevity [10–13]. Intelligence has also been shown to have a substantial influence on school grades [14]. Results from psychometric testing of intelligence are stable across the life span [15, 16]. Intelligence is highly heritable, and the role of genetic factors seems to increase with age [17, 18].

High intelligence leads to better educational attainment (EA) [14]. The direction of this association is however not entirely clear, since ample evidence also indicates that education, especially access to education in adolescence, reliably increases intelligence scores [19–21]. Although EA has traditionally been regarded as an environmental measure, both heritability and environmental factors are thought to affect a significant portion of the variance in EA [22, 23]. Their relative contributions vary across different cohorts [24]. More recent findings describe a high heritability for EA [25], although this inherited phenotype appears to reflect many traits, not just intelligence [25].

Genetically informed studies have revealed a complex web of interactions between intelligence, education, and genetic as well as socioeconomic factors [13, 22–26]. The influence of these factors appears to be particularly dramatic at the extremes of the distribution, suggesting for example that genotypes associated with high EA partially compensate for the disadvantages of children from low-SES families. Children from low-SES families with these favorable genotypes have a more than doubled rate of going to university compared to other children matched on SES [13].

Moreover, parental intelligence is independently associated with child health and behavior, even when adjusting for child IQ and SES [27]. Children with parents who score low on IQ tests have been shown to have an increased risk of conduct, emotional, and attention problems [28]. There is also a clear association between parental education and parental IQ and the EA among their children [29, 30], but it is not known whether the association is equally strong among children with ADHD.

The role of genetic propensity for intelligence in educational performance in those with ADHD has not previously been studied. In this study we aimed to address this knowledge gap, by investigating the link between paternal intelligence as a measure for genetic propensity for intelligence, ADHD, and school grades using regional and national health registers, taking a wide range of potential confounders into account. Specifically, we investigated associations between ADHD and school performance in relation to the fathers’ cognitive performance. Furthermore, we aimed to study whether this association was mediated through ADHD. We also investigated whether ADHD medication use during early and middle childhood influenced the effect of paternal intelligence on school performance for ADHD patients at age 16.

## Methods and materials

### Study population

**Fig. 1:**
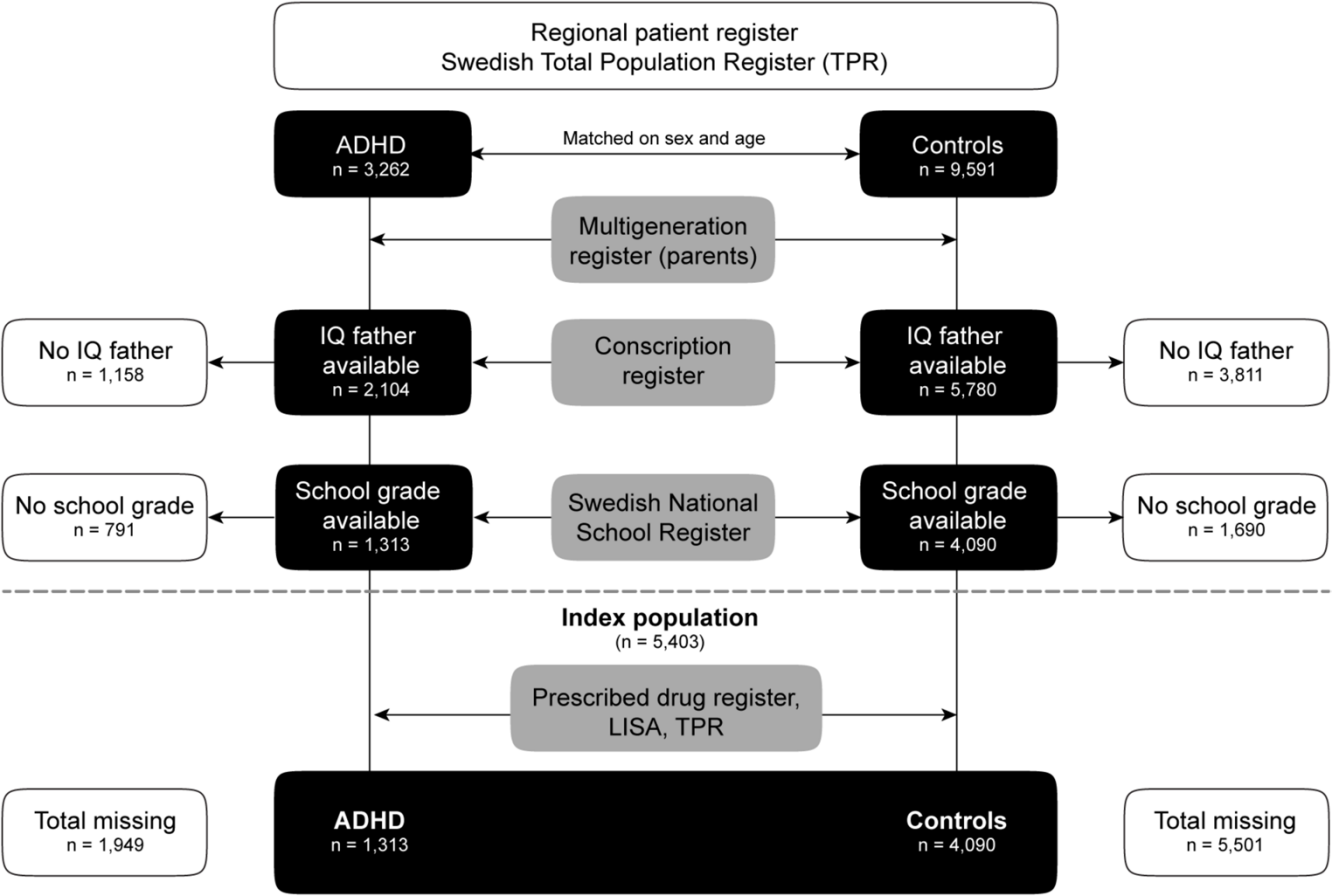
Flowchart showing the selection process for the index population. Abbreviations: Attention deficit hyperactivity disorder (ADHD), the Swedish Total Population Register (TPR), the Longitudinal integrated database for health insurance and labour market studies (LISA)

A flow chart of the sampling of the index population is displayed in Fig. 1. Patients treated for ADHD at the child and adolescent psychiatric clinic (CAP) 1995–2020 were identified using the regional population-based health care record of the County of Östergötland. This contains data on all health care in the region [31, 32], and is since 2006 connected to the electronic medical records in the region [31, 33]. The same health record was used to identify, for each case, three Sex- and age-matched controls, with no ADHD and no visits to the Child and Adolescent Psychiatry clinic (CAP). Information regarding parents and siblings of the study populations was obtained via the Swedish Multi-Generation Register [34]. All data were cross-linked using the Swedish national identity number [35]. National register data were de-identified after extraction to mask participants’ identity and protect their integrity. The study was approved by the Swedish Ethical Review Authority (Dnr 2019–04805).

### Demographics

Demographic information (sex, year of birth and death) was extracted from the Swedish Total Population Register (TPR) containing all Swedish residents born since 1932, and education level from the Longitudinal integrated database for health insurance and labour market studies (LISA) [36]. LISA contains data on education level for all persons aged 16 or older in Sweden since 1990. Using data from LISA we separated EA into three levels: (1) elementary school, (2) secondary school, and (3) college/university level education.

### Fathers’ cognitive performance (intelligence)

Cognitive performance of the fathers, was accessed from the Swedish Defence Conscription and Assessment Agency which holds medical and psychological assessment data on all men born in 1946 or later that have signed up at the Swedish Conscription Agency or the Swedish National Agency for Human Services until 30 June 2010 [37, 38]. Since conscription was mandatory for men born between 1951 and 1987, which include the parental cohort of this study, population coverage for this age group is excellent (approximately 90%) for men, but very low for women. For subsequent birth cohorts (such as the index population), the population coverage has dropped substantially also for men due to changes in Swedish Defence Conscription screening procedures [39]. Cognitive performance was therefore assessed only for the fathers in this study [37]. Attrition analyses showed that subjects with missing paternal IQ were more likely to be born outside of Sweden and younger.

Paternal cognitive performance (paternal IQ) was determined at the time of military conscription, at age 18, with a battery of tests consisting of a total of 200 items, covering logical, verbal, spatial, and theoretical/technical aspects of reasoning. The test battery was revised in 1994. In both tests, individual test scores were summed into a final score known as a stanine scale ranging from 1 (the lowest score) and 9 (the highest) [37].

### School performance

The Swedish school and grading system have been described in detail elsewhere [40]. Swedish primary and lower secondary school consists of nine mandatory school years [41]. During the final semester of the 9th grade (at age 16), students receive a weighted grade in each subject reflecting their overall performance during lower secondary school. The 16 best grades are then summarized to a grade point sum, in the National School Register (NSR). We extracted these grade point sums, hereafter referred to as “school grades at age 16”, for all cases and controls, as a measure of school performance [40, 42].

### ADHD variables

Similar to an earlier study [32], we extracted ADHD diagnoses and medication for the whole population, including parents and siblings, from the National Patient Register (NPR) [43], the Swedish Prescribed Drug Register (PDR) [44], and regional registers [33]. In Sweden, ADHD medications can only be prescribed by a specialist in psychiatry or CAP, thus being a good proxy for ADHD diagnosis. Individuals were considered ADHD-positive if they either had a lifetime diagnosis of ADHD, or had filled at least one prescription of ADHD medication [32]. We included the following medications according to the Anatomical Therapeutic Chemical (ATC) classification system codes: N06BA01 (amphetamine), N06BA02 (dexamphetamine), N06BA04 (methylphenidate), N06BA07 (modafinil), N06BA09 (atomoxetine), N06BA11 (dexmetylfenidat), N06BA12 (lisdexamphetamine), and C02AC02 (guanfacine). As the PDR was introduced in Sweden in July 2005, medication analyses were carried out for patients diagnosed after July 2005. A patient was defined as receiving treatment in any given 6 month-period (starting January 1 or July 1) if they had collected two or more prescriptions of ADHD medication during that time frame [45, 46]. Any patient having received treatment for one or more 6 month-periods was considered medicated. Patients with no such treatment periods were considered unmedicated.

### Statistical analysis

Data were preprocessed with in-house MATLAB scripts (MATLAB Version: R2021b and R2022a) and R Statistical Software (R version 4.3.0). Statistical analyses were done using R Statistical Software and IBM SPSS statistics (Version 27).

Pearson’s chi-square or Wilcoxon rank sum tests were used to test distribution of patient characteristics regarding ADHD status and medication-status for ADHD patients.

Linear mixed models were applied to investigate how paternal IQ was related to child school grades at age 16. We used school grades a as the dependent variable, which was standardized with mean of 0 and SD of 1. Results for standardized school grades are presented as z scores. Paternal IQ, sex, ADHD in the offspring, parent education level and birth year as a non-linear effect were included as fixed effects. Being part of the same first-degree family (parents, siblings) was included as a random effect to account for correlation between siblings. An interaction between paternal IQ and ADHD diagnosis in the offspring was considered, but since it was not significant, it was not included in the final model.

We found a moderate, significant correlation (*r* = 0.58, *p* < 0.001) between paternal IQ and paternal education attainment. Therefore, we performed a sensitivity analysis, where paternal education was removed. In a second sensitivity analysis, based only on participants with ADHD, we examined how paternal IQ affected offspring school grades depending on whether they were medicated or not for their ADHD. As above, sex, education level of the parents and birth year as a non-linear effect were included as fixed effects while being part of the same first-degree family was included as a random effect.

Since having a child with ADHD poses additional demands on parenting and support, we also performed mediation analyses, exploring to what extent the effect of paternal IQ on child school grade was mediated by child ADHD or paternal ADHD.

## Results

**Fig. 2:**
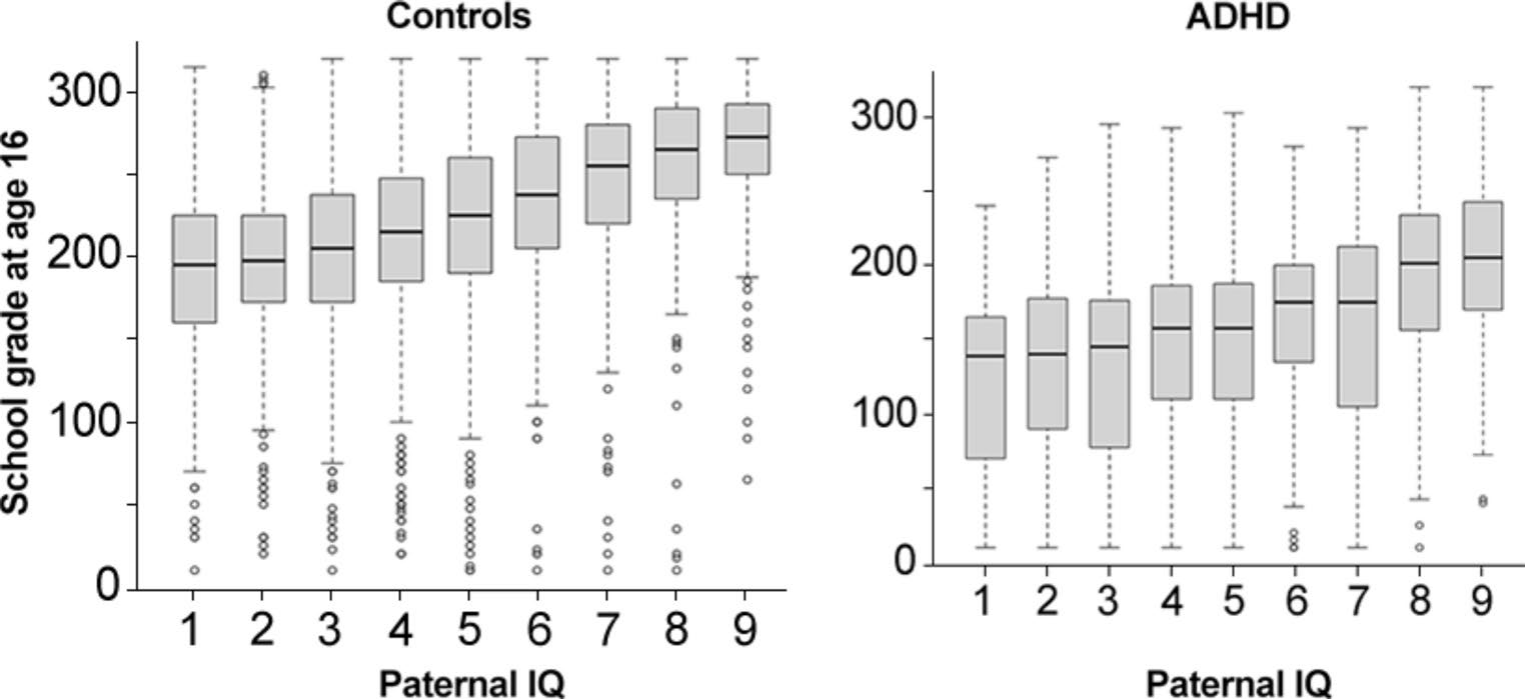
Descriptively shows boxplots of school grades on each level of paternal IQ measured conscription for the control group (left side) and individuals with ADHD (right side)

Study population characteristics are displayed in Table 1. There was a slight overrepresentation of males in the ADHD sample (60.5% vs. 55.4% in controls, *p* = 0.001). The ADHD group showed significantly lower parental education (*p* < 0.001; Table 1) and higher rates of parental ADHD diagnoses (*p* < 0.001; Table 1). Paternal IQ was also lower in those with an ADHD diagnosis compared to controls (*p* < 0.001; Table 1). Patients with ADHD had significantly lower school grades compared to controls (*p* < 0.001; Table 1; Fig. 2).

**Table 1:** Cohort characteristics

	Total	Control	ADHD		*p*-value	
N	5 403	4 090	1 313			
Age in 2019 (median [IQR])	21.0 [18.0, 24.0]	21.0 [18.0, 24.0]	20.0 [18.0, 24.0]	0.068		
Sex (Male)	3 058 (56.6%)	2 264 (55.4%)	794 (60.5%)	0.001		
School grade age 16 (median [IQR])	215.0 [175.0, 260.0]	232.5 [195.0, 270.0]	162.5 [108.8,192.5]	< 0.001		
Father’s IQ (stanine score)						
1	195 (3.6%)	121 (3.0%)	74 (5.6%)	< 0.001		
2	401 (7.4%)	267 (6.5%)	134 (10.2%)			
3	597 (11.0%)	409 (10.0%)	188 (14.3%)			
4	815 (15.1%)	595 (14.5%)	220 (16.8%)			
5	1 130 (20.9%)	871 (21.3%)	259 (19.7%)			
6	875 (16.2%)	681 (16.7%)	194 (14.8%)			
7	654 (12.1%)	541 (13.2%)	113 (8.6%)			
8	465 (8.6%)	385 (9.4%)	80 (6.1%)			
9	271 (5.0%)	220 (5.4%)	51 (3.9%)			
Father’s Highest Education				< 0.001		
Pre-secondary education	526 (9.7%)	377 (9.2%)	149 (11.4%)			
Secondary education	2962 (54.9%)	2178 (53.3%)	784 (59.8%)			
Post-secondary education	1 911 (35.4%)	1 533 (37.5%)	378 (28.8%)			
Missing	4	2	2			
Mother’s Highest Education				< 0.001		
Pre-secondary education	303 (5.6%)	180 (4.4%)	123 (9.4%)			
Secondary education	2 538 (47.0%)	1865 (45.6%)	673 (51.4%)			
Post-secondary education	2 556 (47.4%)	2042 (50.0%)	514 (39.2%)			
Missing	6	3	3			
Father ADHD diagnosis	173 (3.2%)	50 (1.2%)	123 (9.4%)	< 0.001		
Mother ADHD diagnosis	205 (3.8%)	54 (1.3%)	151 (11.5%)	< 0.001		

*Note* A Mann-Whitney *U* test was used to test for group differences in age and school grade, linear-by-linear tests to test father’s IQ and mother’s/father’s highest education, and Pearson’s Chi-squared tests for the other variables.

The children without available paternal IQ were, slightly younger: 17 (IQR 13, 22) versus 19 (IQR 15, 22), slightly more likely to be male (63% vs. 60%) and more likely to be born outside of Sweden. Participants with missing grades are significantly younger than those with available grades: 13(IQR 11,15) vs. 21(IQR 18,24), *p* < 0.001, but showed no difference regarding paternal IQ, *p* = 0.052.

Within the ADHD group (Table 2), boys with ADHD were more likely than girls to have received medication during lower secondary school (*p* < 0.001; Table 2). Those medicated had also slightly lower paternal IQ levels (*p* = 0.034), were more likely to have a father (*p* = 0.0013) or a mother with ADHD (*p* = 0.002) and had slightly lower grades (*p* = 0.008) than unmedicated patients (Table 2).

### Paternal IQ was positively associated with school grade

**Fig. 3:**
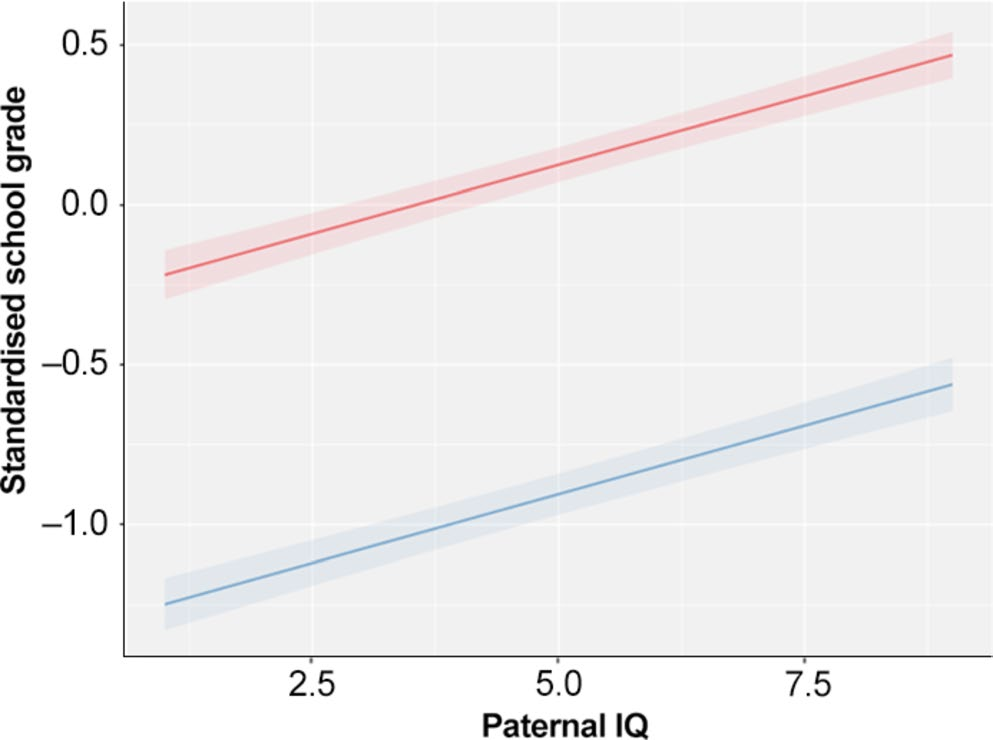
Predicted standardized school grades at age 16 versus paternal IQ at conscription for individuals with ADHD (blue line) and controls (red line). The mixed model contained paternal IQ, sex, parent education level and birth year as fixed effects and family ID as random effect

A linear mixed model showed that higher paternal IQ was significantly associated with a higher standardized school grade in their offspring, even after controlling for sex, age, ADHD, paternal ADHD, and parents´ highest education (Z = 0.09, 95%CI 0.07, 0.10; Table 3). There was no significant interaction between paternal IQ and ADHD diagnosis in the offspring. Although the association was present for both ADHD patients and controls, ADHD patients had on a group level, one standard deviation lower school grade than controls (Z=-1.03, 95%CI -1.08, -0.98 Table 3; Fig. 3).

**Table 2:** Characteristics of ADHD patients by medication status during lower secondary school

	ADHD total	Unmedicated	Medicated	p-value
N	1 220	649	571	
Age in 2019 (median [IQR])	20.0 [18.0, 23.0]	20.0 [18.0, 23.0]	20.0 [18.0, 23.0]	0.006
Sex: Male n (%)	719 (58.9%)	323(49.8%)	396 (69.4%)	< 0.001
School grade age 16 (median [IQR])	165.0 [110.0, 195.0]	167.5 [116.3, 202.5]	157.5 [105.0, 190.0]	0.008
Father’s IQ (stanine score)				0.034
1	63 (5.2%)	34 (5.2%)	29 (5.1%)	
2	124 (10.2%)	64 (9.9%)	60 (10.5%)	
3	177 (14.5%)	88 (13.6%)	89 (15.6%)	
4	200 (16.4%)	89 (13.7%)	111 (19.4%)	
5	240 (19.7%)	133 (20.5%)	107 (18.7%)	
6	188 (15.4%)	110 (16.9%)	78 (13.7%)	
7	104 (8.5%)	59 (9.1%)	45 (7.9%)	
8	76 (6.2%)	41 (6.3%)	35 (6.1%)	
9	48 (3.9%)	31 (4.8%)	17 (3.0%)	
Father’s Highest Education				
Pre-secondary education	137 (11.2%)	71 (11.0%)	66 (11.6%)	
Secondary education	726 (59.6%)	377 (58.2%)	349 (61.1%)	0.237
Post-secondary education	356 (29.2%)	200 (30.9%)	156 (27.3%)	
Missing	1	1	0	
Mother’s Highest Education				
Pre-secondary education	109 (9.0%)	50 (7.7%)	59 (10.4%)	0.072
Secondary education	619 (50.9%)	326 (50.3%)	293 (51.5%)	
Post-secondary education	489 (40.2%)	272 (42.0%)	217 (38.1%)	
Missing	3	1	2	
Father ADHD diagnosis	116 (9.5%)	49 (7.6%)	67 (11.7%)	0.013
Mother ADHD diagnosis	145 (11.9%)	60 (9.2%)	85 (14.9%)	0.002

*Note* A Mann-Whitney *U* tests were used to test for group differences in age and school grade, linear-by-linear tests to test father’s IQ and mother’s/father’s education, and Pearson’s Chi-squared tests for the other variables

**Table 3:** Results from linear mixed models, using paternal intelligence as a predictor for standardized school grades at age 16 in the offspring, controlling for covariates

Analysis (number)	Characteristic	Z scores	95% CI^1^
Main analysis	**Paternal IQ**	**0.09**	**0.07, 0.10**
(*N* = 5393)	ADHD diagnosis	-1.03	-1.08, -0.98
	Sex (Female)	0.28	0.24, 0.32
	Father Highest education	0.09	0.07, 0.11
	Mother Highest Education	0.14	0.12, 0.15
Sensitivity analysis 1 – Excluding paternal education level	**Paternal IQ**	0.12	0.11, 0.13
(*N* = 5397)	ADHD diagnosis	-1.02	-1.07, -0.97
	Sex (Female)	0.27	0.23, 0.31
	Mother Highest Education	0.16	0.15, 0.18
Sensitivity analysis 2– Only in those with ADHD, including medication status as a covariate	**Paternal IQ**	0.08	0.04, 0.11
(*N* = 1216)	ADHD medication status	-0.10	-0.21, 0.00
	Sex (Female)	0.06	-0.05, 0.16
	Father Highest Education	0.07	0.02, 0.12
	Mother Highest Education	0.14	0.10, 0.18

^1^ CI = Confidence Interval; Birth year was included as a non-linear effect in the model

### Sensitivity analyses

In the first sensitivity analysis we excluded paternal education level from the above mixed linear model. Estimates for the effect of paternal IQ on school grades were slightly higher (Z = 0.12, 95%CI 0.11, 0.13; Table 3).

In a second analysis, based only participants with ADHD from our population, we additionally included medication status as a covariate. We still found a positive but somewhat attenuated effect of paternal IQ on school grades (Z = 0.08, 95% CI 0.04, 0.11) and the effect of medication status was not significant in the model (Z=-0.10,95% CI -0.21, 0.00). (Table 3)

### Mediation analyses

#### ADHD does not mediate the effect of paternal IQ on school grades

One research question was whether ADHD in the offspring mediated the effect of paternal IQ on the child’s school grades. However, paternal IQ had no significant effect on child ADHD diagnosis (OR = 0.86, 95% CI = 0.72, 1.04) while controlling for sex and parental highest education levels. Therefore, child ADHD did not meet the precondition for being a mediator and no further analysis was conducted.

### Paternal IQ did not correlate with school grades when accounting for child ADHD

Finally, we examined the hypothesis that paternal IQ affected child school grade through paternal ADHD by replacing child ADHD with paternal ADHD in the mediation analysis. Paternal ADHD was negatively associated with child school grade (Z = -0.46, 95% CI = -0.60, -0.32). However, when child ADHD was also included in the model, the association between paternal ADHD and school grades was no longer significant (Z = 0.00, 95% CI = -0.12, 0.12).

## Discussion

This present study analyzed how paternal IQ and ADHD affect child school grades. In a mixed model adjusting for demographic and SES factors, paternal IQ was, as expected, positively associated with children’s school grades. We found the same association for children with an ADHD diagnosis, albeit school grades were close to one standard deviation lower in those with ADHD compared to controls. ADHD did not serve as an effect modifier or mediator for how paternal IQ affected school grades. The results suggest a main effect of paternal IQ on child grades in both those with and without ADHD diagnosis. The effect was larger when paternal educational level was removed from the model, suggesting that part of the effect of education was through IQ.

Among ADHD patients, medication use during lower secondary school was associated with slightly lower grades, probably due to confounding by indication. The effect of paternal IQ on school grades was still significant when taking medication status into account.

As mentioned, previous studies suggest a complex interplay between ADHD, school grades, SES, intelligence, and genetic factors. ADHD is a highly heritable condition [47], where ADHD in the parent highly increases the risk for ADHD in the offspring. We explored potential mediation of the effect of paternal IQ on school grades through paternal ADHD and through child ADHD. Our results did not support any significant mediation effect, for either paternal ADHD or child ADHD by itself. Paternal IQ was however associated with paternal ADHD, in turn highly associated with child ADHD, which had a significant effect on school grades.

ADHD patients had significantly lower grades compared to controls, in line with earlier research indicating that ADHD negatively affects school performance [40]. We found lower grades for each level of paternal IQ in the crude analysis, and nearly one SD lower grades in our final model, suggesting that adolescents with ADHD underperform in school considering their presumed IQ heritability. The tendency for ADHD patients to receive lower grades is well known [40] and most likely due to a multitude of factors. Partly, ADHD patients seem to score lower in IQ tests [48, 49], and are more likely to have lower than expected estimated IQ scores based on parental IQ [50]. Also in this study, paternal IQ was significantly lower in those with ADHD. On the other hand, disruptive behaviors associated with ADHD have been found to increase levels of student-teacher conflict [49] and seem to negatively influence teacher grading [40]. Further, *g*enetic liability to ADHD has been found to affect EA, independently of cognitive ability [51]. Also, a previous study found that genetic predisposition to higher EA decreased the risk for ADHD diagnosis, an effect which was present independent of the individuals’ cognitive ability [50].

In this paper, ADHD medication was associated with lower school grades for patients. However, the cross-sectional nature of the data on school grades did not permit investigating whether medication may help within-individual grade improvement. Also, medication users were more often male, and males are more likely to get lower school grades [52]. Male sex has also been identified, along with other factors such as symptom severity and psychiatric comorbidities, to increase the likelihood of being prescribed ADHD medication [51, 53–56]. We did not have any data on symptom severity, but it is likely that the lower grades among medication users were an effect of confounding by indication, along with sex differences between medicated and unmedicated patients. Previous studies using repeated measurements of school grades in relation to current medication have found clinically small but significant beneficial outcomes of ADHD medication [40, 57]. In one previous study, medication had large, salutary, effects on children’s academic seatwork productivity and classroom behavior on every single day of the instructional period. However, there was no detectable effect of medication on learning the material taught during instruction [51].

This study has both strengths and weaknesses. Using a large population-based cohort with data linkage from Swedish national and regional registers both for the index group and their relatives provides a comprehensive overview of a clinical population with practically no attrition from follow up, as well as the possibility to explore intergenerational effects of intelligence measures. Some of the limitations are inherent to epidemiological register-based data. Due to coverage issues in the Swedish Defence Conscription and Assessment Agency for women and birth cohorts after 1987, only paternal IQ was analyzed in this study. However, we included maternal EA in the analyses. EA and intelligence highly correlate [58]. As expected, paternal IQ was more likely to be missing in males born outside of Sweden, possibly limiting the generalizability of our findings. We did not have any data on IQ measures in the offspring either, which would have been of great interest to further elucidate the role of intelligence in this material. Children who at the time of data collection had not yet completed lower secondary school naturally lacked data on school grades. Attrition analyses confirmed that participants lacking data on school grades were significantly younger but indicated no significant difference in paternal IQ between groups.

In conclusion, we found that paternal IQ in a large population-based cohort was positively associated with school grades in the offspring, in both those with and without ADHD, even after adjusting for demographic and socioeconomic factors. ADHD patients scored significantly lower grades than controls independent of paternal IQ, indicating ADHD as an important barrier to reaching one’s potential. ADHD medication during lower secondary school was however not associated with higher, but rather with slightly lower grades, probably due to more severe problems in those receiving medication in a naturalistic setting. This paper aids in further understanding of the complex relationship between intelligence and ADHD, both heritable traits affecting school performance in the offspring. Recognizing the complicated relationship between ADHD, intelligence and how they affect school grades, while taking individual demographic and SES factors into account can help clinicians to adapt treatment to the unique preconditions for each patient, facilitating personalized care in clinical practice.

## Data Availability

The data, material and code necessary to reproduce the analyses presented here are not publicly accessible but they can be made available upon reasonable request to the corresponding author, Andrea Markkula.
